# Phosphorylation of calcium/calmodulin-stimulated protein kinase II at T286 enhances invasion and migration of human breast cancer cells

**DOI:** 10.1038/srep33132

**Published:** 2016-09-08

**Authors:** Mengna Chi, Hamish Evans, Jackson Gilchrist, Jack Mayhew, Alexander Hoffman, Elizabeth Ann Pearsall, Helen Jankowski, Joshua Stephen Brzozowski, Kathryn Anne Skelding

**Affiliations:** 1 School of Biomedical Sciences and Pharmacy, Faculty of Health and Medicine, The University of Newcastle, Callaghan, New South Wales, Australia; 2 Priority Research Centre for Cancer, Hunter Medical Research Institute, Faculty of Health, The University of Newcastle, Callaghan, New South Wales, Australia

## Abstract

Calcium/calmodulin-stimulated protein kinase II (CaMKII) is a multi-functional kinase that controls a range of cellular functions, including proliferation, differentiation and apoptosis. The biological properties of CaMKII are regulated by multi-site phosphorylation. However, the role that CaMKII phosphorylation plays in cancer cell metastasis has not been examined. We demonstrate herein that CaMKII expression and phosphorylation at T286 is increased in breast cancer when compared to normal breast tissue, and that increased *CAMK2* mRNA is associated with poor breast cancer patient prognosis (worse overall and distant metastasis free survival). Additionally, we show that overexpression of WT, T286D and T286V forms of CaMKII in MDA-MB-231 and MCF-7 breast cancer cells increases invasion, migration and anchorage independent growth, and that overexpression of the T286D phosphomimic leads to a further increase in the invasive, migratory and anchorage independent growth capacity of these cells. Pharmacological inhibition of CaMKII decreases MDA-MB-231 migration and invasion. Furthermore, we demonstrate that overexpression of T286D, but not WT or T286V-CaMKII, leads to phosphorylation of FAK, STAT5a, and Akt. These results demonstrate a novel function for phosphorylation of CaMKII at T286 in the control of breast cancer metastasis, offering a promising target for the development of therapeutics to prevent breast cancer metastasis.

Breast cancer is the second most commonly diagnosed cancer world-wide^1^. Despite improvements in survival rates, ~1/3 of patients will develop distant metastases^2^, and once breast cancer has metastasised, it is generally thought to be incurable. Recent work has demonstrated that calcium signaling is a controller of breast cancer cell metastasis^3^,^4^,^5^. However, the precise mechanisms involved remain to be fully elucidated.

The multifunctional serine (S)/threonine (T) protein kinase, calcium/calmodulin-stimulated protein kinase II (CaMKII), is one of the major calcium sensors in cells^6^. CaMKII has four isoforms (α, β, γ, δ), one or more of which are expressed in virtually every tissue. As such, CaMKII is involved in controlling a range of cellular processes, including synaptic plasticity and memory consolidation^7^,^8^, vascular smooth muscle polarization and migration^9^, cell proliferation^10^,^11^,^12^, fertilization^13^, and mammary gland lumen formation^14^. Additionally, recent evidence has implicated CaMKII in controlling cancer cell metastasis^15^. Decreasing CaMKII expression in osteosarcoma^16^ and prostate^17^ cancer cells inhibits motility and invasion.

The biological properties of CaMKII are controlled by multi-site phosphorylation and via targeting to specific subcellular microdomains^18^,^19^. When intracellular calcium levels rise, calcium binds to calmodulin, which activates CaMKII, and leads to phosphorylation of CaMKII at T286. Phosphorylation of CaMKII at T286 induces autonomous activation of CaMKII, and sustains CaMKII activity in the absence of an increase in calcium^18^. Phosphorylation of CaMKII at T286 has been implicated in a number of neuronal processes, and has been shown to be essential for the acquisition of fear and spatial learning^7^,^20^,^21^. However, the functions controlled by pT286-CaMKII in non-neuronal cells remain largely unexplored. Recently, CaMKII phosphorylation at T286 has been shown to be increased in a range of cancer types^16^,^22^,^23^, but little is known about the functions of this in cancer cells. Britschgi *et al.*^24^ demonstrated that phosphorylation of CaMKII at T286 contributes to the oncogenic effects of anoctamin-1 (ANO1) in breast cancer. This suggests that phosphorylation of CaMKII at T286 may control processes involved in breast cancer tumourigenesis and progression.

The present study investigated the function of expression and phosphorylation of CaMKII in breast cancer cell proliferation, anchorage-independent growth, migration and invasion. Furthermore, we demonstrate herein that CaMKII phosphorylation at T286 is a prognostic factor for breast cancer, and that increased CaMKII expression and T286 phosphorylation indicates poorer overall and distant metastasis free survival in breast cancer patients. Additionally, pharmacological inhibition of phosphorylation of CaMKII at T286 prevents breast cancer cell migration and invasion. Taken together, our data indicate that T286 phosphorylation of CaMKII controls breast cancer cell migration and invasion, and highlights the potential therapeutic implications of preventing CaMKII phosphorylation at T286 as a new treatment for controlling breast cancer cell metastasis.

## Results

### High CaMKII expression predicts poor breast cancer patient prognosis

**Figure 1:**
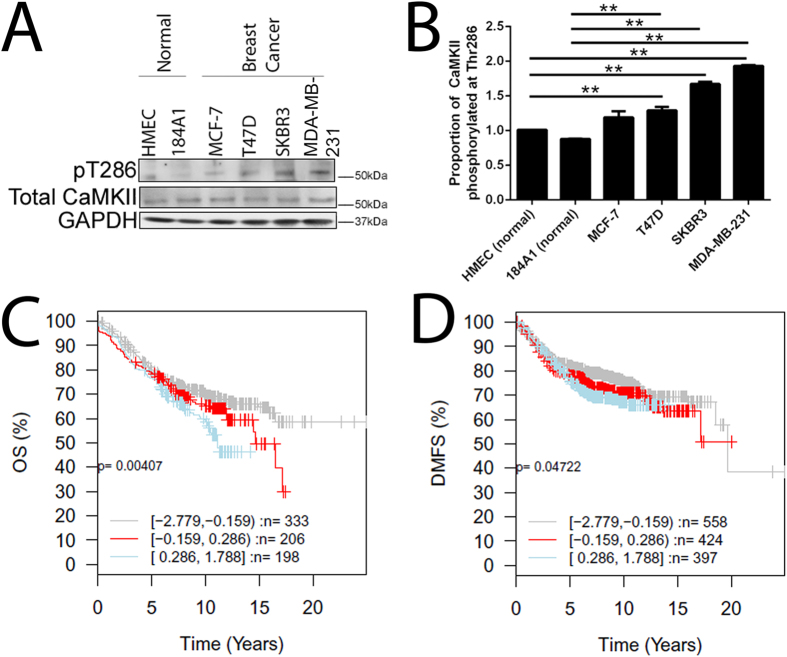
CaMKII phosphorylation at T286 is associated with more aggressive breast cancer, and high *CAMK2* expression predicts worse overall and distant metastasis free survival in breast cancer patients. (**A**) Total endogenous CaMKII and T286 phosphorylation was determined by western blot. GAPDH expression was used as a loading control. Blots are representative of three independent experiments. (**B**) The proportion of CaMKII phosphorylated at T286 was determined by normalising the level of T286 phosphorylation to total CaMKII expression. **denotes statistical significance p < 0.01, as determined by one-way ANOVA. (**C**) Kaplan-Meier curves showing the overall survival [OS] and (**D**) distant metastasis free survival [DMFS] in a publically available 1881-sample breast cancer data set^25^, with high (blue), mid (red) or low (grey) expression of the *CAMK2* family in breast cancer tumours when assessing all tumour subtypes together. P values were computed by a likelihood-ratio test.

CaMKII expression and phosphorylation at T286 was initially examined in a panel of normal and cancerous breast cell lines with varying levels of aggressiveness. Whilst the level of total CaMKII remained relatively unchanged in the panel of breast cell lines examined, breast cancer cells expressed significantly higher levels of pT286-CaMKII when compared to normal breast cell lines (Fig. 1A,B). Furthermore, breast cancer cells that exhibit a more invasive phenotype (MDA-MB-231 and SK-BR-3) possessed the highest proportions of pT286-CaMKII (Fig. 1A,B).

We next assessed whether CaMKII expression is associated with breast cancer patient outcome by examining *CAMK2A*, *CAMK2B*, *CAMK2G* and *CAMK2D* mRNA expression in a publically available 1881-sample breast cancer data set^25^. High *CAMK2* mRNA expression was associated with significantly worse overall (Fig. 1C) and distant metastasis free survival (Fig. 1D) in breast cancer patients when all tumour subtypes were assessed together. These findings were confirmed in an additional 3,935 patient cohort^26^. Additionally, increased *CAMK2* mRNA expression was associated with significantly worse distant metastasis free survival in luminal A (p = 0.016) and triple negative breast cancer (p = 0.006) subtypes in the additional 3,935 patient cohort, but not luminal B or Her-2 subtypes. Furthermore, when the *CAMK2* genes were examined independently, high *CAMK2A* mRNA expression was associated with significantly worse overall (p = 0.01, p = 0.01007, p = 0.01) and distant metastasis free survival (p = 0.05, p = 0.02434, p = 0.01) in Luminal A, Luminal B, and triple negative breast cancer subtypes, respectively. High *CAMK2B* mRNA expression was associated with worse overall and distant metastasis free survival in estrogen receptor positive (ER) tumours (p = 0.00077 and p = 0.0341, respectively). Increased *CAMK2G* mRNA expression was associated with significantly worse overall and distant metastasis free survival in Luminal A (p = 0.0029, p = 0.000217, respectively) and ER positive (p = 0.04, p = 0.0239, respectively) tumours. By contrast, high *CAM2D* mRNA was not associated with significantly worse overall or distant metastasis free survival in the cohorts examined.

**Figure 2:**
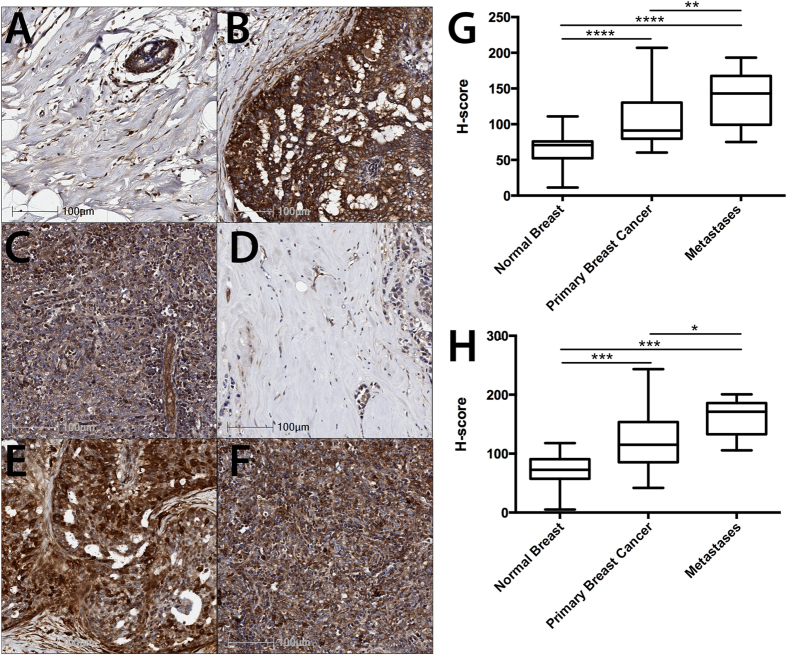
CaMKII expression and phosphorylation at T286 is increased in primary breast cancer and lymph node metastases tissues. (**A,D**) Normal breast, (**B,E**) primary breast cancer, and (**C,F**) lymph node metastases were examined for (**A–C**) total CaMKII and (**D–F**) pT286-CaMKII expression by immunohistochemistry. Staining was quantified and expressed as an H-score. (**G**) Quantification of total CaMKII and (**H**) pT286-CaMKII expression in 70 primary breast cancer, 40 matched normal breast, and 10 lymph node metastases cores. Photomicrographs are representative of each tissue type. *denotes statistical significance p < 0.05, **p < 0.01, ***p < 0.001, ****p < 0.00001, as determined by one-way ANOVA.

To examine the level of CaMKII phosphorylation at T286 in breast cancer tissues and to confirm that total CaMKII is overexpressed in breast cancer tissues at the protein level, CaMKII protein expression and phosphorylation at T286 was examined in 70 breast cancer, 40 matched normal breast, and 10 lymph node metastases patient samples by immunohistochemistry. Total and phosphorylated CaMKII expression was scored on a scale of 0–300, as previously described^27^. In contrast to that observed in the established breast cell lines (Fig. 1A,B), total CaMKII expression was significantly increased in primary breast cancer (Fig. 2B,G; p < 0.00001) and lymph node metastases (Fig. 2C,G; p < 0.00001) when compared to normal breast tissue (Fig. 2A). Furthermore, phosphorylation of CaMKII at T286 was also significantly increased in primary breast cancer (Fig. 2E,H; p < 0.001) and metastases (Fig. 2F,H; p < 0.001) when compared to the normal breast tissue (Fig. 2D). Total CaMKII expression (Fig. 2G; p < 0.01) and phosphorylation at T286 (Fig. 2H; p < 0.05) were further increased in lymph node metastases, when compared to the primary breast cancer samples, providing further evidence for a role of CaMKII in breast cancer cell metastases. Taken together, these data demonstrate that both CaMKII expression and phosphorylation at T286 are increased in breast cancer tissue, as well as lymph node metastases, and may be potentially useful biomarkers to predict patient outcome and likelihood of metastasis. However, the functions controlled by CaMKII in breast cancer cells remain largely unexplored.

### CaMKII promotes migration, invasion and anchorage independent growth of breast cancer cells

As increased *CAMK2* mRNA expression predicts that breast cancer patients will have shorter overall and distant metastasis free survival (Fig. 1), and phosphorylation of CaMKII at T286 was significantly increased in breast cancer and lymph node metastases tissue (Fig. 2), we tested whether CaMKII overexpression or phosphorylation at T286 could alter processes known to be involved in breast cancer cell metastasis. To investigate the role of CaMKII on these cellular processes, a wild-type (WT) form of CaMKII, a T286D phosphomimic mutant form (T286 mutated to D), and a T286V phosphonull mutant form (T286 mutated to V) of CaMKII were transfected into the triple negative MDA-MB-231 line, and the luminal A MCF-7 breast cancer line, and effects on migration, invasion, proliferation, and anchorage independent growth determined. MDA-MB-231 inducibly transfected cells overexpressed FLAG-tagged CaMKII mutants following 24 and 48 h treatment with 2 μg/ml doxycycline, whereas non-induced mutant CaMKII and EV cell lines did not express detectable levels of CaMKII (Supplementary Figure S1A). Furthermore, there was no significant difference between the cell lines overexpressing the 3 CaMKII mutants (p > 0.9868). MCF-7 cells stably transfected with various mutant forms of FLAG-tagged CaMKII expressed ~10-fold greater levels of CaMKII compared to EV cells (Supplementary Figure S1B). Additionally, there was no significant difference between the cell lines overexpressing the 3 CaMKII mutants (p > 0.9928; Supplementary Figure S1B). Importantly, basal phosphorylation of CaMKIIα in these transfected cells was not altered (Supplementary Figure 1A,B).

**Figure 3:**
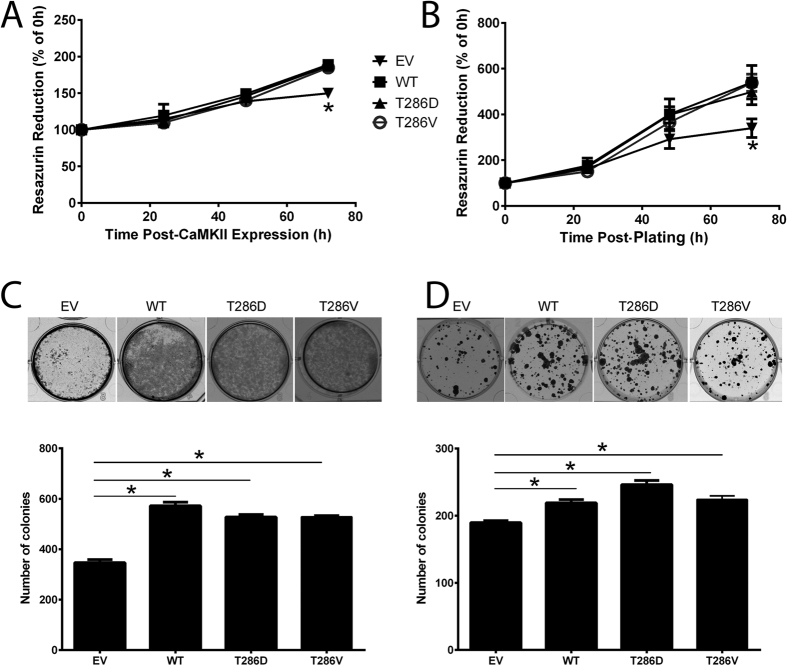
CaMKIIα controls cellular proliferation in breast cancer cells. (**A**) MDA-MB-231 cells inducibly expressing or (**B**) MCF-7 cells stably expressing empty vector (EV), wild-type (WT), T286D, or T286V CaMKII were generated. Cell viability was measured at 0, 24, 48, and 72 h post-CaMKII expression or plating via Cell Titre Blue Assay. *denotes statistical significant difference from EV control cells, as determined by one-way ANOVA (p < 0.05). n = 4. (**C**) MDA-MB-231 and (**D**) MCF-7 cells expressing CaMKII mutants were grown for 8 (MDA-MB-231) or 21 (MCF-7) days. After this time, colonies were stained with 0.5% crystal violet/10% methanol/PBS for 30 mins, and then counted. Photomicrographs are representative of three independent experiments, performed in triplicate. *denotes statistical significant difference p < 0.05, as determined by one-way ANOVA.

We firstly investigated the effects of T286D phosphomimic mutation of CaMKII on the proliferative capacity of MDA-MB-231 and MCF-7 breast cancer cells. As we and others have previously shown^11^,^16^,^22^, overexpression of WT CaMKII significantly increases cell proliferation, when compared to EV control cells, as measured by Cell Titer Blue (Fig. 3A,B) and clonogenic assays (Fig. 3C,D). Additionally, we show that T286D phosphomimic mutation has no further effect on proliferation rates of breast cancer cells *in vitro* (Fig. 3).

**Figure 4:**
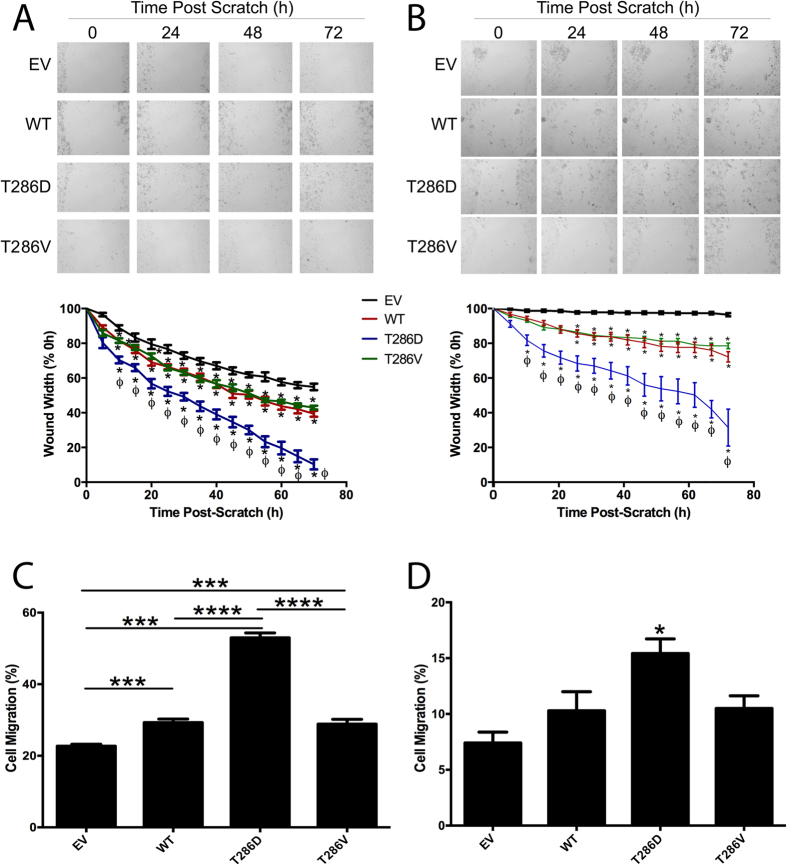
T286D phosphomimic mutation of CaMKII enhances breast cancer cell migration. (**A**) MDA-MB-231 and (**B**) MCF-7 cells expressing empty vector (EV), wild-type (WT), T286D, or T286V CaMKII were grown to confluence, and a wound was made by scratching with a pipette tip. The wounds were photographed hourly for 72 h to measure wound closure over time. Photomicrographs are representative of three independent experiments performed in triplicate. Wound widths are expressed as % of 0 h wound width. *denotes statistical significance from EV control cells (p < 0.05). ^ϕ^denotes statistical significance compared to WT and T286V cells (p < 0.05), as determined by one-way ANOVA. n = 3. (**C**) MDA-MB-231 and (**D**) MCF-7 cells expressing CaMKII mutants were placed in the upper chamber of a Transwell, and allowed to migrate through the uncoated membrane (8 μm pore) for 4 h. n = 3. *denotes statistical significance p < 0.05, ***p < 0.001, ****p < 0.0001 as determined by one-way ANOVA.

We next observed the effects of CaMKII and T286D phosphomimic mutation on breast cancer cell migration. Both MDA-MB-231 (Fig. 4A) and MCF-7 cells (Fig. 4B) overexpressing WT-CaMKII migrated significantly more rapidly than empty vector (EV) control cells (p < 0.01 and p < 0.001, respectively), demonstrating that CaMKII can likely control breast cancer cell migration. Furthermore, MDA-MB-231 and MCF-7 cells overexpressing the T286D phosphomimic mutant form of CaMKII (Fig. 4A,B) migrated significantly more rapidly than the WT and T286V phosphonull forms of CaMKII, indicating that phosphorylation of CaMKII at T286 further increases the migratory capacity of breast cancer cells. As wound healing assays cannot separate migration from proliferation, a transwell migration assay was also performed, and T286D phosphomimic mutation was once again shown to significantly increase MDA-MB-231 (Fig. 4C) and MCF-7 (Fig. 4D) migration, when compared to expression of EV, WT and T286V phosphonull mutant forms of CaMKII. Taken together, this demonstrates that the increased rate of wound closure observed in the T286D phosphomimic mutant overexpressing breast cancer cells (Fig. 4A,B) was not due to increased proliferative capacity, but that T286D phosphomimic mutation of CaMKII increases the migratory capability of breast cancer cells, without altering proliferation rates.

**Figure 5:**
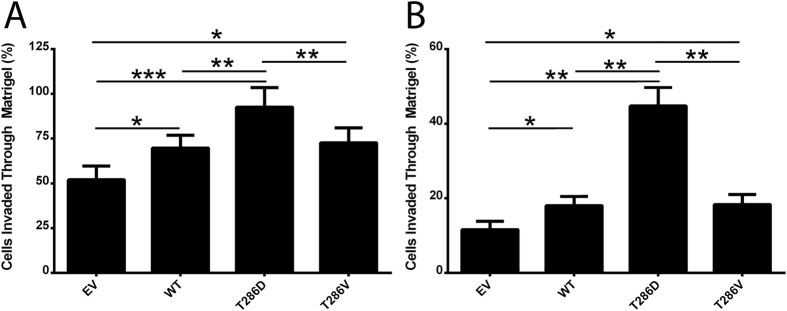
T286D phosphomimic mutation of CaMKII enhances breast cancer cell invasion. (**A**) MDA-MB-231 and (**B**) MCF-7 cells expressing CaMKII mutants were examined for ability to invade through Matrigel plugs. n = 3. *denotes statistical significance p < 0.05, **p < 0.01, ***p < 0.001, as determined by one-way ANOVA.

Similarly, significantly greater numbers of MDA-MB-231 (Fig. 5A) and MCF-7 (Fig. 5B) cells overexpressing WT-CaMKII invaded through Matrigel plugs when compared to control EV cells (p < 0.05, for both), and phosphomimic mutation of T286 further enhanced invasion of both cell lines when compared to WT and T286V expressing cells (Fig. 5A,B; p < 0.001, for both). This is the first evidence identifying cellular functions controlled by pT286-CaMKII in cancer cells.

**Figure 6:**
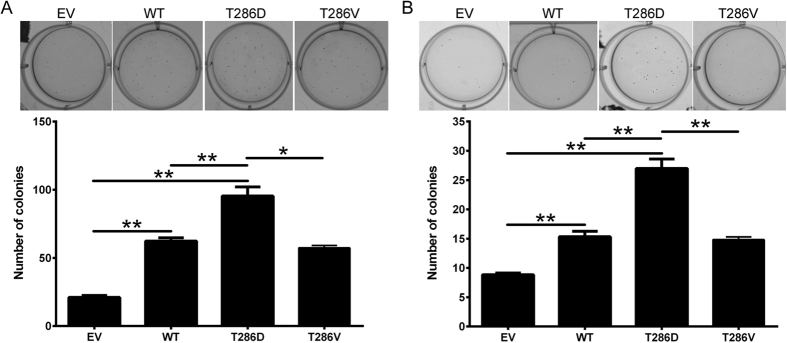
T286D phosphomimic mutation of CaMKII enhances anchorage independent growth. (**A**) MDA-MB-231 and (**B**) MCF-7 cells expressing empty vector (EV), wild-type (WT), T286D, or T286V CaMKII were grown for 14 (MDA-MB-231) or 24 (MCF-7) days in soft agar. After this time, colonies were stained with 0.5% crystal violet/PBS overnight, and then colonies >50 μm were counted using an inverted microscope. Photomicrographs are representative of three independent experiments, performed in triplicate. Results are presented as the number of colonies. *denotes statistical significant difference p < 0.05, **p < 0.01, as determined by one-way ANOVA.

The ability of cancer cells to grow in the absence of adhesion to extracellular matrix (ECM) is closely correlated with tumourigenicity in animal models^28^. WT-CaMKII overexpression significantly increased the ability of both breast cancer cell lines to grow in the absence of ECM (Fig. 6A,B; p < 0.01 for both). Furthermore, overexpression of the T286D phosphomimic form of CaMKII further significantly enhanced the ability of both the invasive MDA-MB-231 (Fig. 6A), and the non-invasive MCF-7 (Fig. 6B) breast cancer cells to grow in a semi-solid medium, when compared to the WT and T286V phosphonull forms of CaMKII. This indicates that phosphorylation of CaMKII at T286 enhances the tumourigenicity of breast cancer cells *in vitro*.

### Pharmacological inhibition of CaMKII decreases breast cancer cell migration and invasion *in vitro*

**Figure 7:**
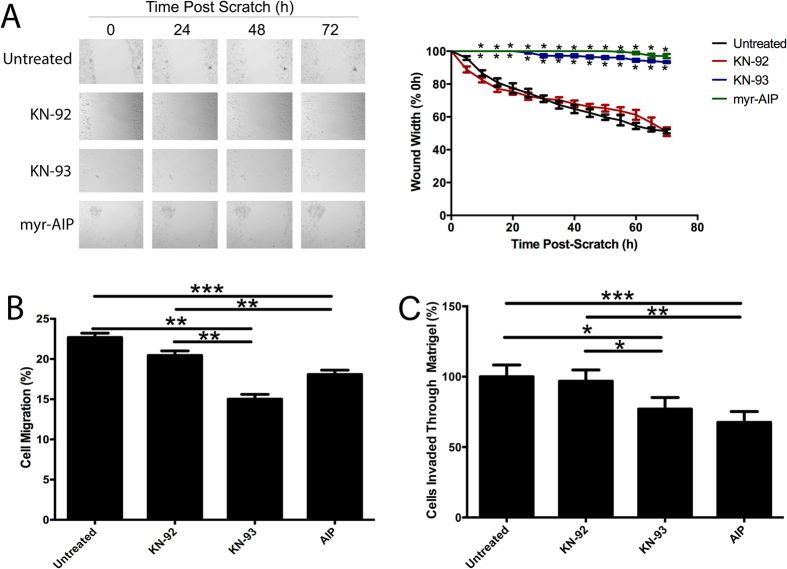
Pharmacological inhibition of CaMKII activity prevents breast cancer cell migration and invasion. Confluent monolayers of parental MDA-MB-231 cells were treated with 20 μM KN-92 or KN-93, or 10 μM myr-AIP for 30 min, and a wound was made by scratching the monolayer with a pipette tip. (**A**) Wounds were photographed hourly for 72 h to measure wound closure over time. Photomicrographs are representative of three independent experiments performed in triplicate. Wound widths are expressed as % of 0h wound width. *denotes statistical significance from untreated cells, p < 0.05, as determined by one-way ANOVA. (**B**) Following this treatment, cells were placed in the upper chamber of a Transwell, and allowed to migrate through the uncoated membrane (8 μm pore) for 4 h. n = 3. **denotes statistical significance p < 0.01, ***p < 0.001, as determined by one-way ANOVA. (**C**) Parental MDA-MB-231 cells were treated with 40 μM KN-92 or KN-93, or 10 μM myr-AIP for 30 min, and the ability of cells to invade through Matrigel plugs was examined. *denotes statistical significance p < 0.05, **p < 0.01, ***p < 0.001, as determined by one-way ANOVA.

Taken together, our data suggest that activation of CaMKII can enhance breast cancer cell motility, invasiveness and tumourigenicity. To investigate whether pharmacological inhibition could potentially decrease breast cancer cell motility and invasion *in vitro*, we inhibited CaMKII activity using two different pharmacological inhibitors with varying mechanisms of action. KN-93 prevents the activation of CaMKII by calcium/calmodulin, but does not inhibit CaMKII that is already autonomously active. However, KN-93 can also inhibit molecules unrelated to CaMKII, such as ion channels. CaMKII specific effects can be determined when the effects of KN-93 are compared to its inactive analogue, KN-92. Myristoylated autocamtide-2-related autoinhibitory peptide (myr-AIP), competes with substrates at the active site of CaMKII, and inhibits activity of CaMKII irrespective of whether CaMKII is autonomously active or not^10^. Both AIP and KN-93, but not KN-92, significantly decreased migration (Fig. 7A,B) and invasion (Fig. 7C) of MDA-MB-231 cells. These findings demonstrate that pharmacological inhibition of CaMKII can significantly inhibit migration and invasion of highly aggressive breast cancer cells *in vitro*.

### Overexpression of T286D-CaMKII leads to increased phosphorylation of FAK, STAT5a, and Akt

CaMKII phosphorylates a range of proteins involved in breast cancer cell metastasis; however, the proteins phosphorylated by pT286-CaMKII in breast cancer cells have not been investigated. We screened 44 proteins involved in breast cancer cell migration and invasion simultaneously using a Phosphoproteome Profiler Array, and confirmed expression/phosphorylation of 7 of the proteins that were significantly altered in cells overexpressing the T286D phosphomimic mutant form of CaMKII, compared to those overexpressing the WT and T286V phosphonull form, to identify proteins/pathways that were altered following T286 phosphorylation.

**Figure 8:**
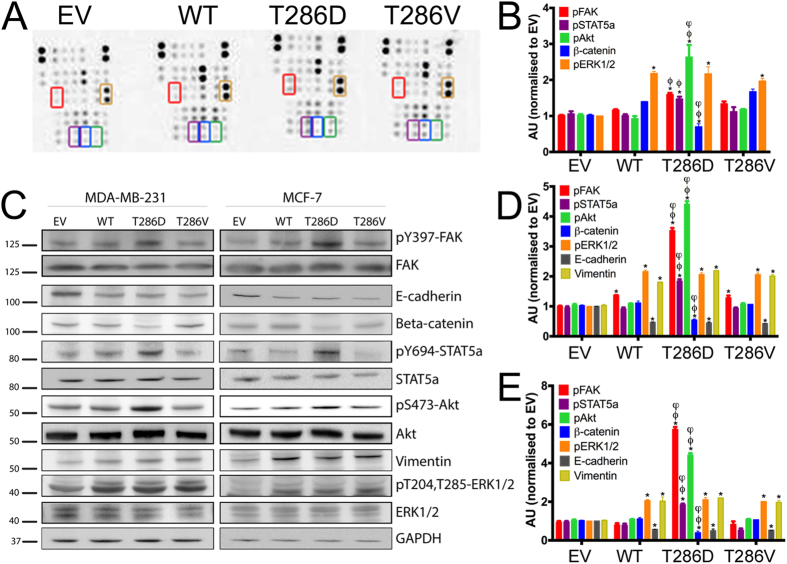
CaMKII overexpression alters the phosphorylation and expression of multiple proteins in breast cancer cells. (**A**) Expression and phosphorylation of 44 proteins were examined by Proteome Profile Human Phospho-Kinase Array following inducible overexpression of empty vector (EV), wild-type (WT), T286D phosphomimic mutant, or T286V phosphonull mutant forms of CaMKII in MDA-MB-231 cells. (**B**) The relative expression and phosphorylation of these proteins were quantitated by densitometric analysis, and expression normalised to that observed in the EV control cells. (**C**) The expression and phosphorylation of 7 proteins found to be differentially expressed/phosphorylated following T286D phosphomimic mutant overexpression in the array were confirmed by Western blot in MDA-MB-231 and MCF-7 cells. Blots are representative of three independent experiments. The relative expression of these proteins were quantitated by densitometric analysis, and expression normalised to that observed in EV (**D**) MDA-MB-231 and (**E**) MCF-7 cells. *denotes statistical significance from EV cells, ^Φ^denotes statistical significance from WT cells, ^φ^denotes significance from T286V expressing cells.

All CaMKII overexpressing breast cancer cells had increased levels of pERK1/2 and vimentin and decreased levels of E-cadherin (Fig. 8A–E), and MDA-MB-231 cells also possessed elevated levels of pFAK (Fig. 8C,D). Furthermore, MDA-MB-231 (Fig. 8C,D) and MCF-7 cells (Fig. 8C,E) overexpressing the T286D phosphomimic mutant form of CaMKII had significantly elevated levels of pFAK, pSTAT5a and pAkt, when compared to EV, WT and T286V phosphonull control cells. This indicates that T286 phosphorylation of CaMKII can lead to increased activation of FAK, STAT5a and Akt.

### Overexpression off T286D-CaMKII may enhance the epithelial-mesenchymal transition in breast cancer cells

The epithelial-mesenchymal transition (EMT) allows epithelial cells to acquire characteristics of mesenchymal cells, such as enhanced motility and invasiveness. As such, EMT plays an important role in the development of metastasis. We next investigated the ability of T286D phosphomimic mutation of CaMKII to alter markers of EMT in breast cancer cells. We found that overexpression of CaMKII in both MDA-MB-231 and MCF-7 cells, significantly increased mesenchymal markers (e.g. vimentin) (Fig. 8C–E), and significantly decreased epithelial markers (e.g. E-cadherin and beta-catenin) (Fig. 8C–E) when compared to EV control cells. Furthermore, overexpression of the T286D phosphomimic mutant resulted in a further significant decrease in beta-catenin and E-cadherin when compared to WT and T286V mutant expressing cells (Fig. 8C–E). This indicates that CaMKII, and specifically pT286-CaMKII, may mediate breast cancer cell motility by initiating the EMT.

## Discussion

Recent studies have demonstrated that CaMKII is involved in controlling osteosarcoma and gastric cancer cell invasion and migration^16^,^22^, and lung cancer tumourigenicity^29^, and we have previously implicated CaMKII in breast cancer cell proliferation^11^,^30^. However, the role of CaMKII phosphorylation in cancer cell invasion and migration has not previously been explored. Our data presented herein show that breast cancer cell invasion, migration and anchorage independent growth can be enhanced by phosphorylation of CaMKII at T286, and that if this phosphorylation is prevented using pharmacological inhibitors, this invasion and migration can be prevented. Furthermore, our data indicates that the cellular effects of pT286-CaMKII may be mediated by initiating the EMT, and by activation of FAK, STAT5a and Akt.

Increased *CAMK2* mRNA was associated with significantly worse overall and distant metastasis free survival in the breast cancer patients examined (Fig. 1), indicating that high *CAMK2* mRNA is a potentially novel biomarker for breast patient outcome. However, precisely how increased *CAMK2* is producing these adverse effects is unknown.

Whilst there are four isoforms of CaMKII, overexpression of WT-CaMKIIα has previously been shown to control osteosarcoma^16^ and gastric^22^ invasion and migration. We also found that increased *CAMK2A* mRNA expression was associated with significantly worse distant metastasis free survival in Luminal A and triple negative breast cancer patients. Therefore, we utilised CaMKIIα and Luminal A and triple negative cell lines for our overexpression experiments performed in this study.

Daft *et al.*^16^ and Liu *et al.*^22^ have previously examined the role of CaMKIIα in osteosarcoma and gastric cancer cell metastasis, however no previous investigation of the function of CaMKII phosphorylation in these processes has been performed. Herein, we show that pT286-CaMKII is increased in primary breast cancers and their associated lymph node metastases, when compared to normal breast tissue (Fig. 2).

Our findings are consistent with the previous studies examining the role of CaMKII in invasion and migration, and suggest that CaMKIIα can control breast cancer cell migration and invasion. CaMKII is a multifunctional kinase that has been shown to phosphorylate several proteins involved in invasion and migration, including FAK^31^, matrix metalloproteinase-9 (MMP-9)^22^, and stathmin^32^.

Our results suggest that it is not just CaMKII expression that is important for controlling cancer cell invasion and migration, but rather that phosphorylation of CaMKII at T286 can further enhance these processes (Figs 4 and 5), indicating that abundant autonomous activation of CaMKII may result in significantly worse outcome for breast cancer patients by increasing breast cancer cell motility and invasiveness. Additionally, we show that pharmacological inhibition of CaMKII activity using two distinct inhibitors (KN-93 and myr-AIP) prevents breast cancer cell migration and invasion (Fig. 7). Taken together, these findings indicate that CaMKII inhibitors may be useful for preventing breast cancer metastasis.

To investigate the molecular mechanisms underlying the pro-metastatic properties of pT286-CaMKII in breast cancer, over 44 proteins known to be important in cancer cell metastasis were examined. Increased levels of pFAK, pSTAT5a, and pAkt (Fig. 8) were noted in cells expressing high levels of the T286D phosphomimic mutant form of CaMKIIα. FAK is a well known promoter of tumour progression and metastasis^33^, and has previously been shown to be activated by CaMKII in murine fibroblast cells^34^. The Akt-mTOR signalling pathway is known to promote tumourigenesis and metastasis of breast cancer^35^, and CaMKII can activate Akt in vascular smooth muscle cells^36^. STAT5a was first identified as a “mammary gland factor”^37^, but its role in breast cancer progression has not been fully elucidated, as its activation has been shown to play a role in mammary tumour initiation^38^, and to maintain differentiation and suppress the EMT^39^. However, a role for active STAT5 in metastasis of other cancers has been established, and active STAT5a promotes prostate cancer invasion and migration^40^. Whilst CaMKII does not phosphorylate STAT5a at the site examined in this study, a FAK-mediated pathway can control STAT5 activation in leukaemia cells^41^. Furthermore, FAK^42^ and Akt^43^ are essential for inducing EMT in hepatocytes and cancer cells, respectively. Taken together, our data suggest that pT286-CaMKII may enhance breast cancer metastasis via a FAK and Akt-dependent mechanism.

Our findings have identified a new mechanism for controlling breast cancer cell metastasis, specifically phosphorylation of CaMKII at T286. Autonomously activated CaMKII enhances breast cancer metastasis, and pharmacological inhibition of CaMKII activity prevents breast cancer cell invasion and migration *in vitro*. These data provide evidence that CaMKII activation is a novel target for the treatment of breast cancer metastasis.

## Materials and Methods

### Cell Lines and Generation of Mutant CaMKII Expressing Cells

MCF-7 (ATCC HTB-22), SKBR-3 (ATCC HTB-30), and T47D (ATCC HTB-133) cells were purchased from the ATCC (Manassas, VA, USA) and maintained in RPMI-1640, supplemented with 2 mM glutamine and 10% heat-inactivated fetal calf serum (FCS; Sigma-Aldrich, Castle Hill, NSW, Australia). MDA-MB-231 (ATCC HTB-26) and 184A1 (ATCC CRL-8798) were purchased from the ATCC and maintained in DMEM, supplemented with 15 mM HEPES, 2 mM glutamine, and 10% FCS. Human Mammary Epithelial Cells (HMEC) were purchased from Life Technologies, and maintained in HMEC basal serum free medium supplemented with HMEC Supplement and 0.05 mg/ml bovine pituitary extract. All cell culture reagents were purchased from Life Technologies (Mulgrave, VIC, Australia) unless otherwise noted.

MDA-MB-231 cells inducibly expressing FLAG-tagged-CaMKIIα mutants (empty vector [EV], wild-type [WT], T286D phosphomimic, T286V phosphonull), and MCF-7 cells stably expressing CaMKIIα-FLAG-tagged mutants were generated as previously described^11^,^30^.

### CaMKII Inhibitors

2-[N-(2-hydroxyethyl)]-N-(4-methoxybenzenesulfonyl)]amino-N-(4-chlorocinnamyl)-N-methylbenzylamine [KN-93], and 2-[N-(4-methoxybenzenesulfonyl)]amino-N-(4-chlorocinnamyl)-N-methylbenzylamine, phosphate [KN-92] (Calbiochem, Kilsyth, VIC, Australia) were dissolved in dimethyl sulphoxide (DMSO), and myristoylated-AIP [autocamtide-2-related inhibitory peptide; myristoyl-Lys-Lys-Ala-Leu-Arg-Arg-Gln-Glu-Ala-Val-Asp-Ala-Leu] (Biomol, Hamburg, Germany) was dissolved in distilled water. Stock solutions were stored at −20 °C.

### Bioinformatics Analysis of *CAMK2* Expression

Bioinformatic analysis of the four genes encoding CaMKII (*CAMK2A, CAMK2B, CAMK2G, CAMK2D*) were assessed individually and as a group using data from the gene expression based outcome for breast cancer online algorithm (GOBO)^25^. GOBO is a web based analysis tool that utilises 11 publically available Affymetrics U133A gene expression data curated from 1,881 breast cancer patients with associated stage, grade, nodal status and intrinsic molecular classification^25^. Association of outcome was investigated for the total patient cohort, irrespective of subset, with relapse free survival, distant metastasis free survival, or overall survival as end points, and no time-dependent censoring.

Retrospective Kaplan–Meier relapse free survival analyses of 3,935 human patients with invasive breast cancer were performed using an updated version of the previous Kaplan-Meier plotter database^26^. Patients were divided into two groups according to the median mRNA expression levels of the four genes encoding CaMKII. Each percentile of expression between the lower and upper quartiles was computed, and the best performing threshold was used as cutoff for the Kaplan–Meier analyses.

### Tissue microarray

Tissue microarrays (TMAs) were purchased from SuperBioChip Laboratories (Seoul, South Korea), and consisted of 70 breast cancer cores, 10 matched lymph node cores, and 40 matched normal breast tissue samples. The tissues were stained for CaMKII expression and phosphorylation at T286 using a rabbit monoclonal antibody against total CaMKII (1:85; Abcam, Cambridge, MA, USA) or pT286-CaMKII (1:100; Abcam), using the Ventana Discovery Ultra automated system (Ventana Medical Systems Inc., Tucson, AZ, USA). The slides were dewaxed by heating at 69 °C for 24 mins, and antigen retrieval was performed using a high pH buffer at 99 °C for 32 min. Endogenous peroxidase activity was blocked with Inhibitor CM (Ventana Medical Systems) for 8 mins, and then primary antibody was added for 1 h at 35 °C. The samples were then incubated with Discovery OmniMap anti-rabbit HRP for 32 mins (Ventana Medical Systems). The slides were developed with 3,3′-diaminobenzidine tetrahydrochloride substrate and counterstained with haematoxylin (Ventana Medical Systems). Negative controls were included by using non-immune rabbit sera and omitting the primary antibody incubation step. Slides were scanned using an Aperio Scanscope (Leica Biosystems, North Ryde, NSW, Australia), and the H-score calculated. This method assigns an IHC H-score to each patient on a continuous scale of 0–300, based on the percentage of cells at different staining intensities, which was determined using HALO software (Indica Labs, Corrales, NM). The H-score was calculated as follows:

1 × (% of lightly stained cells) + 2 × (% of intermediate stained cells) + 3 × (% of darkly stained cells)^27^.

### Soft Agar Assay

Anchorage independent growth of transfected MDA-MB-231 and MCF-7 cells was measured by plating 10,000 cells in semisolid culture media (1.2% methylcellulose; Sigma-Aldrich) supplemented with 20% FCS (and 2 μg/ml doxycycline for MDA-MB-231 cells), on a layer of 0.7% agar in growth medium. Fresh growth medium was added weekly. At the end of 14–24 days incubation, colonies were stained with 0.05% crystal violet/PBS solution overnight at room temperature and colonies >50 μm were counted. Assays were plated in triplicate, and three independent experiments were performed.

### Tumourigenic Assay

Tumourigenicity of transfected MDA-MB-231 and MCF-7 cells was measured by plating 1000 cells in 6-well plates in growth medium (with 2 μg/ml doxycycline for MDA-MB-231 cells). At the end of 8–21 days incubation, colonies were stained with crystal violet for 30 min and counted. Assays were plated in triplicate, and three independent experiments were performed.

### Cell Titre Blue Assay

Transfected MDA-MB-231 and MCF-7 cells were seeded (1 × 10^4^/well). At various times post plating (stable MCF-7 cells) or post-CaMKII induction (2 μg/ml doxycycline for the MDA-MB-231 cells), proliferation was assessed using the Cell Titer-Blue Cell Viability Assay (Promega, Alexandria, NSW, Australia), as per manufacturer’s instructions. Assays were plated in quadruplicate, and three independent experiments were performed.

### Scratch Migration Assay

Scratch wound migration assays were conducted using transfected MCF-7 and MDA-MB-231 cells, and parental MDA-MB-231 cells following treatment with CaMKII inhibitors. Parental MDA-MB-231 confluent cell monolayers in 24-well plates were preincubated for 30 mins with 20 μM KN-92 or KN-93, or 10 μM myr-AIP. Confluent monolayers of transfected MDA-MB-231 (pre-treated with 2 μg/ml doxycycline for 24 h) and MCF-7 cells in 24-well plates were scratched with a P200 tip. Scratched monolayers were washed twice with sterile phosphate buffered saline (PBS). Medium was replaced with serum free media (with 2 μg/ml doxycycline for MDA-MB-231 cells), and wounds were photographed hourly for 72 h using a humidified, automated live cell microscope at 37 °C/5% CO_2_ (Carl Zeiss, North Ryde, NSW, Australia). Cell migration was analysed using AxioVision v4.9.1 software (Carl Zeiss) to measure the size of the wound, by averaging three individual measurements of wound size for each wound at each time point. Results from three independent experiments with three replicates per experiment were pooled, and data were expressed as percentage of the wound width (compared to 0 h).

### Transwell Migration Assay

The migratory properties of transfected MCF-7 and MDA-MB-231 cells, and parental MDA-MB-231 cells treated with CaMKII inhibitors were investigated using a Cultrex BME Cell Invasion Assay Kit (R&D Systems, Gymea, NSW, Australia), as per the manufacturer’s instructions. Parental MDA-MB-231 cells were serum starved for 24 h, and then pretreated for 30 min with 20 μM KN-92 or KN-93, or 10 μM myr-AIP prior to plating. MDA-MB-231 cells inducibly expressing CaMKII were pre-treated with 2 μg/ml doxycycline (in serum free media) for 24 h to induce CaMKII expression prior to plating. MCF-7 cells were serum starved for 24 h prior to plating. Transwell chambers (8 μM pore) were left uncoated. Cells (5 × 10^4^ cells/chamber in serum free media) were added to the top chambers, and 10% FCS was added to the lower chambers. The cells were allowed to migrate for 4 h, after which time, medium was removed from the top and bottom chambers. Migrated cells on the underside of the chamber were detected by incubating with Calcein-AM dissolved in 1 x Cell Dissociation Buffer (final concentration: 0.8 μM) for 1 h. Fluorescence of the samples was determined at λ_excitation_ 485 nm and λ_emission_ 520 nm using an ELISA plate reader (FLUOStar Optima; BMG Labtech, Mornington, VIC, Australia). The number of cells that migrated through the BME coat were calculated using a standard curve, per manufacturer’s instructions. Results from three independent experiments with three replicates per experiment were pooled.

### Transwell Invasion Assay

The invasive properties of transfected MCF-7 and MDA-MB-231 cells, and parental MDA-MB-231 cells treated with CaMKII inhibitors were investigated as described above, with the following modification. Prior to the addition of cells, transwell chambers (8 μM pore) were coated with 1 x basement membrane extract (BME) solution in coating buffer overnight at 37^o^ C, before excess buffer was removed.

### Western Blotting

Stably transfected MCF-7 cells or inducibly transfected MDA-MB-231 cells that had been treated with 2 μg/ml doxycycline for 24–48 h were lysed as previously described^44^. Cell lysates (10–20 μg) were separated using 10% SDS-polyacrylamide gel electrophoresis (PAGE), and then transferred to nitrocellulose membranes^44^. The primary antibodies used were as follows: GAPDH (1:2,000; BioVision, Milpitas, CA, USA), FAK (1:1,000; Abcam), pY397-FAK (1:1,000; Abcam), ERK1/2 (1:1,000; Abcam), pT204/T285-ERK (1:1,000; Abcam), Akt (1:1,000; Abcam), pS473-Akt (1:1,000; Abcam), E-cadherin (1:1,000; Abcam), β-catenin (1:4,000; Abcam), pY694-STAT5a (1:1,000; GeneTex, Redfern, NSW, Australia), STAT5a (1:1,000; GeneTex), vimentin (1:1,000; Abcam). Blots were scanned with a Fujifilm LAS-3000 Imaging System and analysed with MultiGauge Software (Fujifilm, Brookvale, NSW, Australia).

### Proteome Profiler Human Phospho-Kinase Array

The simultaneous phosphorylation of 44 proteins in transfected cells were detected using the Proteome Profiler Human Phospho-Kinase Array Kit (R&D systems, Abingdon, UK), as per manufacturer’s instructions.

### Data Analysis

All statistical analyses were conducted using GraphPad Prism software V6.0 (GraphPad, San Diego, CA, USA). Comparisons between mutants were made using one-way analysis of variance (ANOVA), with a Bonferonni post-test. The Kaplan-Meier survival analysis was calculated using the Cox proportional hazard model. All data is presented as the mean ± standard error of the mean (SEM) for the number of replicates (n).

## Additional Information

**How to cite this article**: Chi, M. *et al.* Phosphorylation of calcium/calmodulin-stimulated protein kinase II at T286 enhances invasion and migration of human breast cancer cells. *Sci. Rep.* **6**, 33132; doi: 10.1038/srep33132 (2016).

## Supplementary Material

Supplementary Information
