# Supplementary material for: Phosphorylation of calcium/calmodulin-stimulated protein kinase II at T286 enhances invasion and migration of human breast cancer cells
Source: Sci Rep. 2016 Sep 8;6:33132. doi: 10.1038/srep33132 (PMC5015093; doi:10.1038/srep33132)
Supplement: Supplementary Information [file srep33132-s1.doc]

**Phosphorylation of calcium/calmodulin-stimulated protein kinase II at T286 enhances invasion and migration of human breast cancer cells**

Mengna Chi^1,2^, Hamish Evans^1^, Jackson Gilchrist^1^, Jack Mayhew^1^, Alexander Hoffman^1^, Elizabeth Ann Pearsall^1,2^, Helen Jankowski^1,2^, Joshua Stephen Brzozowski^1,2^, Kathryn Anne Skelding^*1,2^

**SUPPLEMENTAL METHODS**

**Western Blotting**

Stably transfected MCF-7 cells or inducibly transfected MDA-MB-231 cells that had been treated with 2µg/ml doxycycline for 24 – 48h were pelleted and then resuspended in lysis buffer (1% NP-40/Tris-buffered saline, 2mM ethylenediaminetetraacetic acid [EDTA], 20µM sodium orthovanadate, 50mM sodium fluoride, cOmplete Protease Inhibitor Cocktail; Roche, Castle Hill, NSW, Australia) for 20 min at 4°C. Lysates were generated as described in Materials and Methods. Cell lysates (10–20µg) were separated using 10% SDS-polyacrylamide gel electrophoresis (PAGE), and then transferred to nitrocellulose membranes, as previously described [^1^](#_ENREF_1). The primary antibodies used were as follows: total pan CaMKII (1:5,000; Millipore, North Ryde, NSW, Australia), phospho-T286-CaMKII (1:1,000; Abcam), FLAG (M2; 1:1,000; Sigma-Aldrich), actin (AC-15; 1:50,000; Sigma-Aldrich). Blots were scanned with a Fujifilm LAS-3000 Imaging System and analysed with MultiGauge Software (Fujifilm, Brookvale, NSW, Australia).

**Supplementary Figure S1. Expression of CaMKIIα constructs in MDA-MB-231 and MCF-7 transfected cells.** (A) MDA-MB-231 cells inducibly expressing empty vector (EV), wild-type (WT), T286D, or T286V CaMKII were generated. At various times post treatment with 2µg/ml doxycycline (0, 24, and 48 h), cells were lysed, and expression of FLAG-tagged CaMKII, and pT286 was determined by western blot. Actin expression was used as a loading control. (B) MCF-7 cells were stably transfected with EV, WT, T286D, or T286V CaMKII constructs. Cells (1 x 10^6^) were lysed, and expression and T286 phosphorylation of CaMKII and actin determined by western blot. Blots are representative of three independent experiments.

**
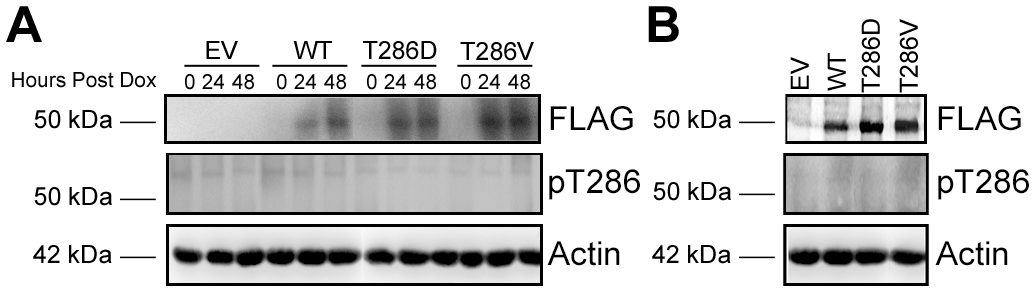
**

**Supplementary Figure S1.**
